# Using a Nature-Based Virtual Reality Environment for Improving Mood States and Cognitive Engagement in Older Adults: A Mixed-Method Feasibility Study

**DOI:** 10.1093/geroni/igac015

**Published:** 2022-03-17

**Authors:** Saleh Kalantari, Tong Bill Xu, Armin Mostafavi, Angella Lee, Ruth Barankevich, Walter R Boot, Sara J Czaja

**Affiliations:** 1 Department of Human Centered Design, Cornell University, Ithaca, New York, USA; 2 Department of Psychology, Florida State University, Tallahassee, Florida, USA; 3 Division of Geriatrics and Palliative Medicine, Center on Aging and Behavioral Research, Weill Cornell Medicine, New York, New York, USA

**Keywords:** Cognitive impairment, Mood, Nature, Virtual garden, Virtual reality

## Abstract

**Background and Objectives:**

Exposure to nature and nature-based imagery has been shown to improve mood states and stave off cognitive decline in older adults. Even “micro-doses” of natural scenery can provide beneficial effects in situations where more extensive interactions with nature are not feasible. In the current study, we evaluated the use of virtual reality (VR) for delivering interactive nature-based content with the goal of prompting active engagement and improving mood states in older adults.

**Research Design and Methods:**

The researchers developed a novel VR environment that combined 360-degree videos of natural areas and botanical gardens with interactive digital features that allowed users to engage with aspects of the environment. We recruited 50 older adults to try out this VR environment and measured changes in mood states and attitudes toward VR from before versus after the sessions. We controlled for variables such as age, education level, and exposure to nature in everyday life, and we looked for differences in responses to the VR among participants with cognitive impairments (CIs) versus without, and participants with physical disabilities versus without.

**Results:**

The findings indicated significant improvements in “good” mood and “calm” mood dimensions after exposure to the VR, as well as improvements in attitudes toward the technology. These positive outcomes were significantly greater for participants with physical disabilities compared to those without disabilities. No differences were found in the responses of participants with CIs versus those without. Exit interviews provided a variety of helpful suggestions about ways to improve the VR equipment design and content to meet the needs of an older adult population.

**Discussion and Implications:**

The study demonstrates that VR can provide a cost-effective, noninvasive, and nonpharmaceutical approach for improving the lives of older adults in both clinical and recreational settings, particularly when real-world access to nature is limited.

**Translational Significance:** This research examined an innovative way to let older adults experience the benefits of natural scenery and engagement with nature, by delivering 360-degree immersive videos and a digitally designed “virtual garden” through virtual-reality (VR) technology. We found that after trying out the experience, our participants reported more positive mood states and more positive views of VR. While many older adults understandably regard new technologies with suspicion, the study showed that VR can serve as an inexpensive, noninvasive, and nonpharmaceutical approach for improving the quality of life, particularly for individuals whose everyday exposure to nature may be limited.

## Background and Objectives

Millions of older adults in the United States suffer from cognitive impairment (CI), a condition that can result from a variety of underlying causes and is associated with both memory-related and nonmemory-related losses of function, and that often progresses to more severe forms of dementia (Lee et al., 2014; Roberts & Knopman, 2013). In addition to the direct impacts for individuals who are living with CI, family members and caretakers often confront burdens associated with the effects of the condition (Lee et al., 2014; Park & Shin, 2016). CI may also contribute to the high numbers of older adults who struggle with loneliness (Perissinotto et al., 2012; Yang & Victor, 2011) and depression (Broadhead et al., 1990; Fiske et al., 2009; Kennedy et al., 1991; Unützer et al., 1997). In recent years, the effects of the coronavirus disease 2019 pandemic have added to the challenges of older adults living with CI, as the need for restrictions on social contact has reduced opportunities for engagement and travel, as well as limiting the accessibility of in-person healthcare interventions (Macdonald & Hülür, 2021; Paananen et al., 2021). Fortunately, there are a wide range of options available to help mitigate the effects of CI. These interventions are rapidly shifting away from an exclusive focus on in-person care to incorporate technological aids, telemedicine, video-based socializing, and other care and activity programming grounded in electronic media (Steinman et al., 2020).

### Exposure to Nature as an Intervention for Individuals With CI

Engaging with natural environments has been linked to improvements in cognitive functioning. For example, researchers have found that increases in neighborhood vegetation are associated with slower cognitive decline (de Keijzer et al., 2018), a lower prevalence of Alzheimer’s disease (Brown et al., 2018), and overall better cognitive abilities among older adults (Prohaska et al., 2009). Improvements in physiological factors such as immune function, blood pressure, and heart rate have also been linked to nature exposure, which may help to explain its impact on cognitive functioning (Alfonsi et al., 2014). Many researchers have also focused on the mental health benefits of nature, particularly in regard to reductions in anxiety, depression, apathy, and negative mood states, as a hypothesized mechanism for its positive impact on cognitive function (e.g., Besser, 2021; Chalfont et al., 2020; Heath, 2004; Kaplan, 1995; Rounds et al., 2020). The benefits of nature exposure for improving mood states have been widely documented in diverse contexts, ranging from wilderness areas to residential streets to urban green spaces and gardens (Aspinall et al., 2015; Brooks et al., 2017; Gidlow et al., 2016; Kondo et al., 2018; Mayer et al., 2009; Song et al., 2013; Ulrich, 2002; Van Den Berg & Custers, 2011). Because benefits for mood states and cognitive function have been found even in “micro-doses” of nature such as viewing urban gardens, the incorporation of green spaces into the built environment could potentially have widespread impacts on the prevalence and severity of CI. However, the rapid increase in urbanization globally has left many city-dwellers without access to such spaces (Kalantari & Shepley, 2020; UN DESA, World Urbanization Prospects, 2019). Furthermore, even when they do have proximal access to nature, older adults are often limited in their ability to experience such spaces by obstacles such as limited mobility, pain, and fear of falling (Appel et al., 2020).

### Virtual Reality and Nature

When direct access to nature is not available on a regular basis, researchers and healthcare practitioners have turned to supplementary approaches for obtaining some of its benefits, such as window views (Chang & Chen, 2005; Kaplan, 2001; Raanaas et al., 2016), nature-oriented artwork and murals (Diette et al., 2003), nature videos (Kahn et al., 2008), and virtual reality (VR) immersion (Gorini et al., 2010; Moyle et al., 2018). Like all such approaches, the use of VR has liabilities, particularly in regard to its lack of tactile engagement and its inability to fully replicate the deep complexity and material interconnectedness of actual organic environments. At the same time, however, the use of VR headsets—or, less commonly, full-room projection systems (Annerstedt et al., 2013; Wang et al., 2020)—helps to create an immersive experience that reduces distractions and can grant reprieve from negative features or limitations of the immediate real-world environment (Furman et al., 2009; Gorini et al., 2010). Recent studies also showed similarity in eye blink rate and heart rate, and electroencephalography frequency band-power in comparison between the real-world and the virtual environment during cognitive tasks (Kalantari et al., 2021). VR is also a very cost-effective intervention in comparison to the construction of real-world green spaces and/or supervised travel to those spaces (Kalantari & Neo, 2020; Kalantari et al., 2022; Yeo et al., 2020), and it can promote active engagement through the incorporation of game-like features. While the use of VR should not be seen as a substitute that replaces the imperative toward real-world biophilic and sustainable design, it can nonetheless provide an effective supplementary approach, especially in situations where direct access to nature is not immediately feasible.

The most basic form of nature exposure through VR focuses on experiencing immersive video footage, such as vistas, beaches, forests, and gardens (Anderson et al., 2017; Appel et al., 2020; Gorini et al., 2010; Moyle et al., 2018; Nukarinen et al., 2020; Riva et al., 2020; Yeo et al., 2020). Such experiences may involve a static user position or a predetermined movement path, while allowing users to freely look around and view different portions of the surroundings through the use of head-motion-tracking technologies. This type of VR is relatively simple and low-cost to construct, as all it really requires is multidirectional video-recording equipment (Nukarinen et al., 2020). Studies using these methods have found improvements in relaxation, mood, and alertness during and after the VR experiences of nature (Anderson et al., 2017; Moyle et al., 2018). However, this type of experience is still quite passive, as the videos generally lack opportunities for engagement and exploration. Appel et al. (2020) noted that once participants had experienced a VR video-footage scene in its entirety, they showed little interest in immediately viewing it again or looking for additional details. As such, the level of cognitive engagement was not much different from watching a flat-screen video. While the passive viewing of VR nature scenes does have demonstrated benefits for mood improvement and stressreduction, the technology is available to create more interactive components and thus prompt greater active engagement. Rendering programs can be used to create digital scenes and elements that are programmed to respond to users’ actions, and that can allow users to fluidly move through the environment following a path of their own choosing. This approach has not yet been widely applied or studied in the context of mental health or cognitive function.

## Virtual Reality and Older Adults

VR technologies have previously been tested with older populations and found to be an effective tool for such individuals. A review by D’Cunha et al. (2019) found strong evidence that older adults who used VR tended to experience mood improvements and reductions in apathy, and that the participants reported enjoying the experiences. Manera et al. (2016) found that participants in a reminiscence-focused intervention using VR reported greater satisfaction with the experience compared to those who completed a similar paper-based activity. Appel et al. (2020) gathered feedback from older adults, some of whom had been diagnosed with mild CI, on the experience of viewing nature scenes presented using VR. The responses to these experiences were quite positive, with a high reported comfort level and enjoyment, and no reported negative side effects. Researchers have also found that attitudes toward VR among older adults significantly improved after experiencing the technology for the first time (Huygelier et al., 2019). Nonetheless, negative impacts of VR have occasionally been reported in the wider literature, including experiences of cybersickness, headaches, and very rarely, the triggering of epileptic seizures (Keshavarz et al., 2018; Seifert & Schlomann, 2021). It is important to be aware of these potential side effects when making use of the technology.

### Study Goals and Hypotheses

In the current study, the researchers created and tested a VR platform oriented toward older adults, which included a selection of 360-degree nature videos as well as a digital “VR Garden” in which users were able to interact with plants and animals and engage in gardening activities. The research presented here focused on the feasibility, usability, and likely adoption of these VR tools. We employed a variety of quantitative measurement instruments (discussed in more detail later) to evaluate mood, engagement, and perceptions of the technology. We also gathered qualitative/interview data to obtain further insights about the participants’ experiences. The current study did not address the long-term impacts of the VR garden on cognitive function; this will be evaluated in future work. The current study was designed to test four primary hypotheses:

*Hypothesis 1*: Short-term exposure to the Virtual Garden will be associated with improvements in mood states.
*Hypothesis 2*: Short-term exposure to the Virtual Garden will be associated with improvements in attitudes toward VR technologies.
*Hypothesis 3*: Changes in mood states and changes in attitudes toward VR technology will be similar between participants who have mild cognitive impairment vs. those who do not have cognitive impairment.
*Hypothesis 4*: Positive changes in mood states and in attitudes toward VR technology will be significantly more pronounced among participants who have physical disabilities, compared to those who do not have physical disabilities.

## Research Design and Methods

### Design of the Virtual Environment

The researchers designed the Virtual Garden using modeling, UV-mapping, and mesh optimization in Autodesk 3ds Max. The design documents were imported into Epic Games’ Unreal Engine 4, and blueprint visual scripting along with C++ scripting was then used to create the interaction components. We included four modules within the overall VR environment: a tutorial, a set of 360-degree nature videos, an interactive garden, and a gardening game.

**Figure 1.**
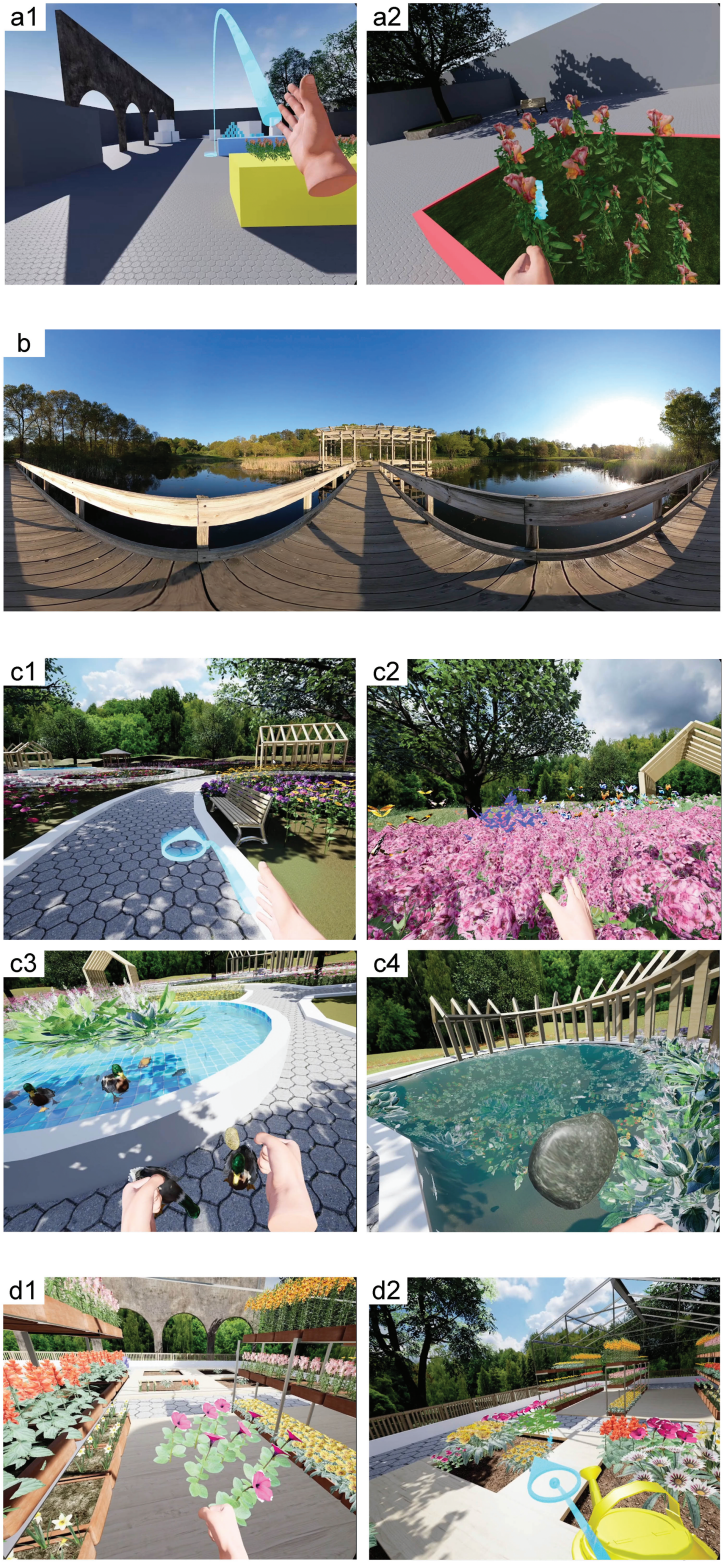
Screenshot captures of the VR environment. (A) The tutorial helped participants to learn the VR controls and to become familiar with the equipment. (B) The video module allowed participants to view 360-degree footage of local natural areas. (C) The interactive tools allowed participants to engage in activities such as (C1) walking along pathways, (C2) touching the flowers, (C3) feeding the ducks, and (C4) throwing rocks into a pond. (D) The interactive components also allowed participants to develop their own cultivation areas in which they could arrange and water various types of flowering plants. VR = virtual reality.

The *tutorial* module was designed as a learning experience to help participants become familiar with the VR system and its navigational and interaction controls. In this module, the participants encountered a relatively small (40 × 40 meters) open space surrounded by walls. The space included several distinct interaction areas, highlighted in different colors, where participants could read instructional text and learn and practice with the VR controls. Upon entering the area, the participants were asked to learn how to move themselves to each interaction site and then to follow the instructions and engage with the virtual objects located at each site. All of the skills needed to navigate the Virtual Garden were practiced in the learning trial, including moving from place to place at different speeds, interacting with textual elements, and grabbing and moving objects (Figure 1A).

The *nature videos* module presented a passive restorative experience. We used a GoPro Fusion 360 camera to record nine short videos of natural areas and botanical gardens in Tompkins County, NY. The resolution of these videos was set to 5k and then later downsized to match the VR display capability, as discussed below. The videos were between 30 and 45 s in length, for a total combined duration of about 5 min. They were rendered using Adobe Premiere Pro CC and the GoPro FX Reframe plugin, and then projected onto a sphere as an environment within the Unreal Engine. Participants were limited to a static position when viewing these videos, but they were able to freely look around and view different perspectives on the 360-degree environment. The videos included recorded sounds from the respective natural areas and gardens (Figure 1B).

The *interactive virtual garden* was an artificial environment created by the researchers to reflect experiences of nature while promoting active engagement. The design of the engagement components drew heavily on existing evidence-based design guidelines for therapeutic gardens (Marcus & Sachs, 2013). Participants were able to experience the virtual garden in a fairly passive fashion by simply “walking” through it (using hand-held controllers to initiate or pause motion along designated paths) and looking around to observe the trees, plants, flowers, ponds, fountains, and benches along the way. They could also engage in interactions with various elements of the garden, for example, by touching the flowers (in response butterflies would come out of some areas), feeding the ducks in the pond, and throwing rocks into the pond. The vibration functions of the hand-held controllers as well as natural sound recordings were used as feedback for interactions and to help improve the garden’s realism (Figure 1C).

The *gardening game* was the most interactive aspect of the virtual experience. In this module, participants were able to create a garden layout and plant and water a variety of flowers to achieve their own desired aesthetic. Eight cultivation areas were included in the overall interactive garden, each containing multiple planting spots. Participants were able to choose from different flowering plants (*Gazania*, *Antirrhinum*, *Salvia*, *Narcissus*, *Petunia*, *Papaver*, *Lilium*, and *Eschscholzia*) that they could move from a storage area to one of the planting spots. To minimize any physical difficulties related to bending down, the planting process was designed as an automatic drop from a hand icon into the soil, complete with a rewarding sound when the planting was accomplished. The participants could then use a watering can to nurture their garden, which resulted in the growth of the plants over time (Figure 1D). The goal of this minigame within the virtual garden was to promote engagement and the maintenance of cognitive skills (attention, memory, and executive control; Anguera et al., 2013).

**Figure 2.**
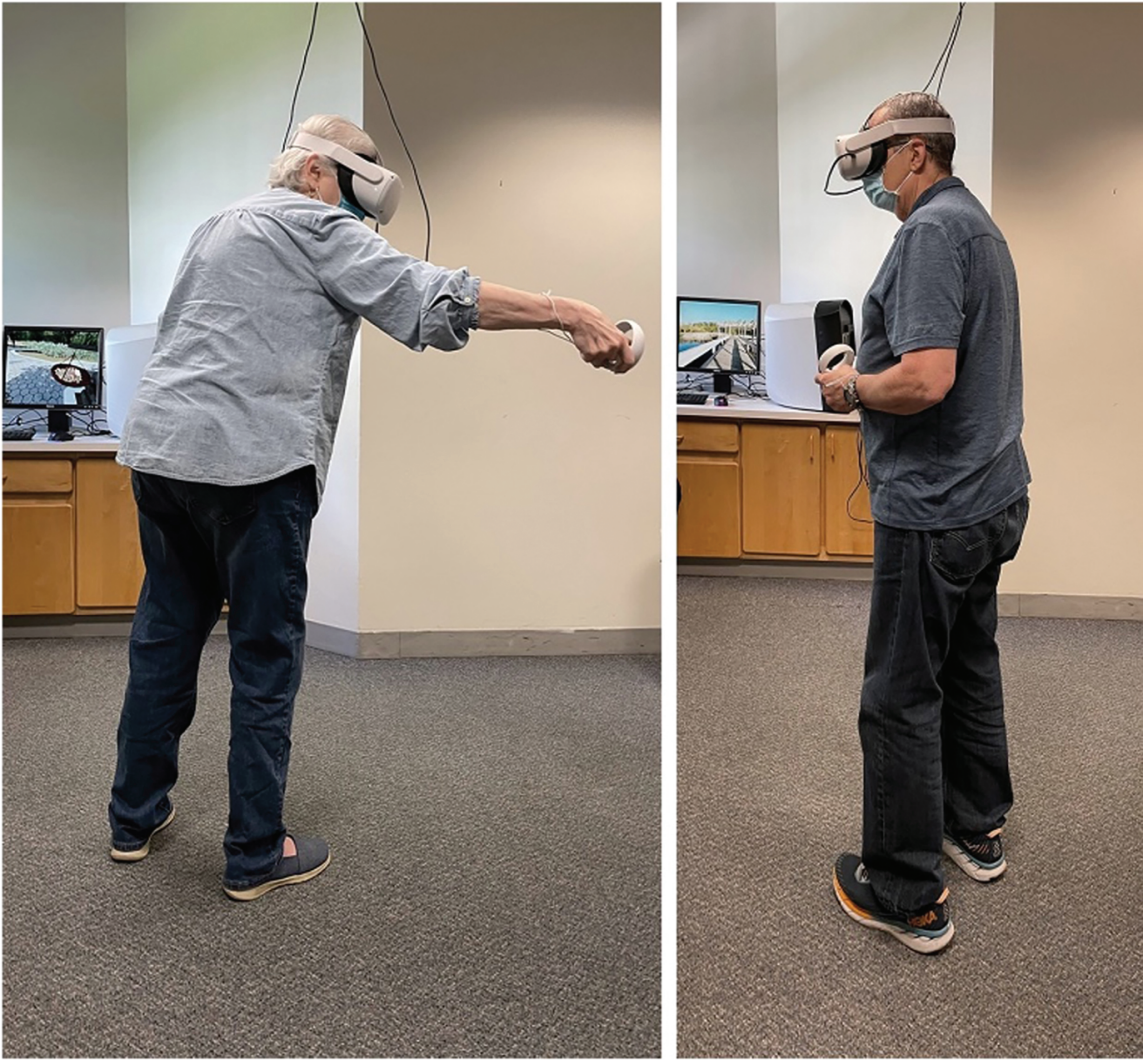
Examples of study participants interacting with the Virtual Garden (images used with the participants’ permission). Interactions could take place from either a standing or seated position, depending on each participant’s preference.

To present the VR environments to the participants, we used a consumer version of the Oculus Quest 2 headset with Oculus Touch controllers for the right and left hands, for all sessions and all participants. The resolution of the Oculus Quest 2 is 1,832 × 1,920 per eye, with a 90 Hz refresh rate. The head-mounted display has a 6-degrees-of-freedom inside-out tracking system, which uses external references in the real-world environment to precisely determine the direction of the user’s gaze. The participants could choose to be seated or standing during all interactions, and motion within the VR environment was enacted through the hand-held controllers. The camera height within the environment was determined automatically based on each participant’s eye-height above the floor (Figure 2).

### Participants

An a priori analysis of the required sample size for this study was conducted using the G*Power software tool, which indicated that approximately 51 participants were needed for a robust statistical power (0.80) in the comparisons that we tested. We used a convenience sampling approach to recruit 52 participants in the Tompkins County, NY area, using fliers in local senior living centers and calls for volunteers on community e-mail lists. All of the participants provided informed consent to participate in the study. The recruitment procedures and overall study protocols were reviewed and approved by the Institutional Review Board at Cornell University. We screened the participants for exclusion criteria focused on a history of seizures, epilepsy, severe motion sickness, and the use of implanted medical devices such as pacemakers. One participant was excluded from the study upon discovering that she used a pacemaker, and a second participant withdrew after experiencing a heightened confusion episode during the experiment. Data are reported for the remaining 50 participants.

### Study Procedure

After providing informed consent, each participant was asked to fill out a demographic questionnaire. This survey form was completed remotely and on the participant’s own time. Each participant was then invited to schedule an individual experimental session at the study site in Ithaca, NY. Upon arriving for the session, each participant filled out additional questionnaires to assess current mood states, attitudes toward VR technology, and cognitive capabilities (Supplementary Material). The researchers then introduced the participant to the hand-held controllers and assisted with donning the VR headset. Two researchers were present at each session, one of whom focused on providing technological assistance and support for the participant, and the other who focused on conducting questionnaires and making empirical observations.

**Figure 3.**
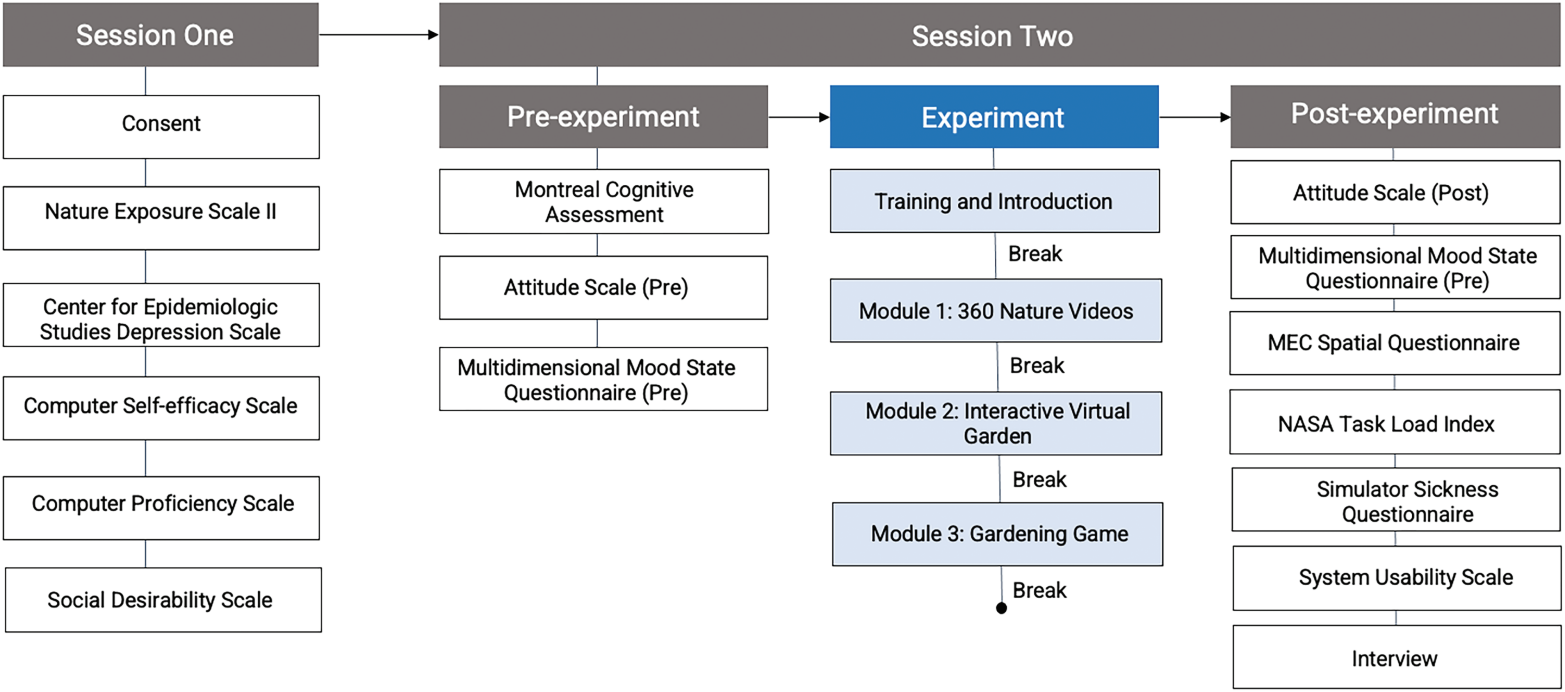
Schematic representation of the study protocol, including the order of test instruments used in the study.

After donning the headset each participant completed the tutorial module. This required approximately 5 min, with very little variation in completion times or observed frustration levels among the different participants. They were then asked to remove the headset and take a 2-min break, during which they engaged in small-talk and informal feedback with the researchers. Next, the participant was asked to put the headset back on and passively view the nature videos component for approximately 5 min, engage with the interactive aspects of the garden for approximately 10 min, and then spend approximately 8 min cultivating their own section of the garden. Between each of these segments, the participant again temporarily removed the headset and took a 2-min break. Finally, each participant was asked to fill out a third questionnaire, which repeated the previous assessments of mood and attitudes toward VR, as well as assessments of immersion and cybersickness. The researcher then conducted a semistructured exit interview with each participant to gather qualitative feedback about their experiences. With the participants’ permission, these interviews were recorded and then transcribed for thematic analysis. Upon completing the session, each participant was presented with a small gift certificate and a visual snapshot of the virtual garden that they had created. The full study protocol is presented schematically in Figure 3.

### Measurement Tools

The study used a wide array of measurement instruments derived from prior research. Some of these instruments were incorporated into the questionnaire forms, while others were completed observationally by the researchers. Details about these measurement tools and how they were used are included in the Supplementary Material. The instruments used in the study were the *Nature Exposure Scale II* (Wood et al., 2019), the *Center for Epidemiologic Studies Depression Scale* (Hann et al., 1999), the *Computer Self-Efficacy Scale* (Barbeite & Weiss, 2004), the *Computer Proficiency Scale* (Boot et al., 2015), the *Montreal Cognitive Assessment* (Nasreddine et al., 2005), the *Multidimensional Mood State Questionnaire* (Steyer et al., 1997), the *Acceptance of Head-Mounted Virtual Reality in Older Adults Scale* (Huygelier et al., 2019), the *MEC Spatial Questionnaire* (Vorderer et al., 2004), the *NASA Task Load Index* (Hart & Staveland, 1988), and the *Simulator Sickness Questionnaire* (Kennedy et al., 1993).

## Results

### Descriptive Statistics and Potential Confounding Variables

The average age of the participants was 67.98. The majority were female (74%), White (90%), and had completed at least a Bachelor’s-level education (80%). A broad range of income levels were represented. The average score on the *Center for Epidemiologic Studies Depression Scale* was 12.36 (*SD* = 6.88), and 13 out of the 50 participants exceeded the threshold for being classified as likely to be experiencing depression. The average score on the *Computer Self-Efficacy Scale* was 56.86 (*SD* = 8.16), and on the *Computer Proficiency Scale* it was 25.91 (*SD* = 3.39), both of which indicate a moderately high level of familiarity with information technology among the participants. The participants’ level of exposure to nature in everyday life was quite high, with an average score on the *Nature Exposure Scale II* of 26.6 (*SD* = 4.04) out of a total possible 30 points. The majority of the participants (80%) had not been previously diagnosed with a physical disability. The average score on the *Montreal Cognitive Assessment* was 25.88 (*SD* = 2.81), with 24 participants (48%) exceeding the threshold for likely having a CI. Overall, the demographic variables were well-distributed among those participants with CI versus those without, and well-distributed among disability versus nondisability participants (Table 1).

**Table 1.** Demographic Characteristics of the Study Participants

Characteristic	Total (*n* = 50)	Non-CI (*n* = 26)	CI (*n* = 24)	Nondisability (*n* = 40)	Disability (*n* = 10)
Age in years, *M* (*SD*)	67.98 (4.85)	67.46 (4.88)	68.53 (4.87)	67.60 (4.89)	69.18 (4.77)
Gender, *n* (%)					
Male	13 (26.0)	4 (15.4)	9 (37.5)	12 (30.0)	1 (10.0)
Female	37 (74.0)	22 (84.6)	15 (62.5)	28 (70.0)	9 (90.0)
Ethnicity, *n* (%)					
White	45 (90.0)	23 (88.5)	22 (92.7)	36 (90.0)	9 (90.0)
All other ethnicities (including mixed ethnicity)	5 (10.0)	3 (11.5)	2 (8.33)	4 (10.0)	1 (10.0)
Marital status, *n* (%)					
Single	7 (14.0)	4 (15.4)	3 (12.5)	5 (12.5)	2 (20.0)
Married	25 (50.0)	13 (50.0)	12 (50.0)	22 (55.0)	3 (30.0)
Divorced	11 (22.0)	6 (23.1)	5 (20.8)	9 (22.5)	2 (20.0)
Separated	3 (6.00)	1 (3.85)	2 (8.33)	1 (2.50)	2 (20.0)
Widowed	4 (8.00)	2 (7.69)	2 (8.33)	3 (7.50)	1 (10.0)
Education level, *n* (%)					
Doctorate degree	9 (18.0)	4 (15.4)	5 (20.8)	6 (15.0)	3 (30.0)
Professional degree	3 (6.00)	3 (11.5)	2 (8.33)	3 (7.50)	0 (0.00)
Master’s degree	18 (36.0)	18 (69.2)	7 (29.2)	17 (42.5)	2 (20.0)
Bachelor’s degree	10 (20.0)	10 (38.5)	4 (16.7)	5 (12.5)	4 (40.0)
Associate degree	3 (6.00)	3 (11.5)	2 (8.33)	3 (7.50)	0 (0.00)
Some college credit/no degree	7 (14.0)	7 (26.9)	4 (16.7)	6 (15.0)	1 (10.0)
Total annual income, *n* (%)					
Less than $50,000	12 (24.0)	6 (23.1)	6 (12.5)	7 (17.5)	3 (30.0)
$50,000–$69,999	12 (24.0)	7 (26.9)	5 (20.8)	10 (25.0)	0 (0.00)
$70,000–$89,999	10 (18.0)	5 (19.2)	4 (16.7)	8 (20.0)	2 (20.0)
$90,000–$119 999	6 (12.0)	4 (15.4)	2 (8.33)	6 (15.0)	4 (40.0)
Greater than $120 000	3 (6.00)	1 (3.85)	2 (8.33)	3 (7.50)	0 (0.00)
Prefer not to answer	8 (16.0)	3 (11.5)	5 (20.8)	6 (15.0)	1 (10.0)
Montreal Cognitive assessment,^a^*M* (SD)	25.88 (2.81)	27.96 (1.43)	23.62 (2.10)	26.15 (2.26)	24.50 (4.17)
Disability, *n* (%)					
Yes	10 (20.0)	4 (15.4)	6 (25.0)	—	—
No	40 (80.0)	22 (84.6)	18 (75.0)	—	—

*Note:* CI = cognitive impairment; *SD* = standard deviation.

^a^Scores range between 0 and 30.

Descriptive statistics for all of the measurement instruments used in the study are presented in Table 2. There was a very low amount of cybersickness reported, with an average score of 2.70 (*SD* = 3.51) on the *Simulator Sickness* instrument. The results for the *NASA Task Load Index* (indicating stress and frustration) also indicated a low average score of 2.12 (*SD* = 0.52). We conducted a correlation test with Holm’s correction to search for potential relations among some of the nonhypotheses measurements (Table 3). Scores on the *Computer Self-Efficacy Scale* and the *Computer Proficiency Scale* were found to be significantly correlated with each other, which is expected since these are both are measurements of familiarity with information technology. We also found that *Nature Exposure* scores (regular experiences of real-world nature) were significantly correlated with changes in attitudes to technology during the experiment (*t* = 2.847, *p* = .007), and to a lesser extent, with the spatial presence self-location (SPSL) component of the *MEC* questionnaire (*t* = 1.906, *p* = .064). The participants’ education level was significantly correlated with changes in spatial presence possible actions (SPPA) during the experiment (*t* = −2.230, *p* = .031), and to a lesser extent, with SPSL (*t* = −1.889, *p* = .066).

**Table 2.** Descriptive Statistics for Data Collected During the VR Sessions

Measure	Total (*n* = 50)	Non-CI (*n* = 26)	CI (*n* = 24)	Nondisability (*n* = 40)	Disability (*n* = 10)
Simulator sickness	2.70 (3.51)	2.73 (3.41)	2.67 (3.68)	2.67 (3.81)	2.80 (1.99)
NASA task load index	2.12 (0.52)	2.05 (0.50)	2.19 (0.55)	2.11 (0.53)	2.15 (0.51)
Social desirability	9.08 (2.11)	9.15 (2.05)	9.00 (2.21)	9.20 (2.10)	8.60 (2.17)
Spatial presence: self-location (SPSL)	34.52 (5.66)	35.23 (4.36)	33.75 (6.82)	33.70 (5.98)	37.80 (2.20)
Spatial presence: possible actions (SPPA)	30.52 (4.67)	30.96 (4.28)	30.04 (5.11)	30.07 (4.94)	32.30 (2.98)
System Usability Scale	72.06 (5.04)	70.73 (4.92)	73.50 (4.85)	71.72 (5.33)	73.40 (3.53)
Mood: awake/tired					
Presession	46.70 (6.62)	45.62 (7.16)	47.88 (5.91)	46.65 (6.67)	46.90 (6.76)
Postsession	47.24 (7.94)	47.54 (8.45)	46.92 (7.51)	46.73 (8.04)	49.30 (7.59)
Delta	0.54 (6.89)	1.92 (6.22)	-0.96 (7.39)	0.07 (6.77)	2.40 (7.40)
Mood: calm/nervous					
Presession	47.44 (5.68)	46.96 (5.49)	47.96 (5.95)	47.52 (5.98)	47.10 (4.53)
Postsession	50.78 (6.20)	51.19 (5.10)	50.33 (7.29)	50.02 (6.55)	53.80 (3.26)
Delta	3.34 (6.17)	4.23 (6.02)	2.38 (6.32)	2.50 (6.28)	6.70 (4.57)
Mood: good/bad					
Presession	49.40 (5.70)	48.96 (5.85)	49.88 (5.62)	49.08 (5.96)	50.70 (4.57)
Postsession	51.08 (5.88)	51.27 (5.34)	50.88 (6.52)	49.98 (5.89)	55.50 (3.27)
Delta	1.68 (4.78)	2.31 (4.84)	1.00 (4.72)	0.90 (4.60)	4.80 (4.39)
Attitude toward VR					
Presession	70.18 (10.73)	70.65 (10.42)	69.67 (11.26)	69.42 (9.79)	73.20 (14.09)
Postsession	75.00 (9.57)	76.00 (8.90)	73.92 (10.32)	73.47 (9.56)	81.10 (7.16)
Delta	4.82 (8.76)	5.35 (9.35)	4.25 (8.23)	4.05 (8.29)	7.90 (10.33)

*Notes:* Standard deviations are presented in parentheses. More information about the measures can be found in Supplementary Material. CI = cognitive impairment; VR = virtual reality.

**Table 3.** Pairwise Correlation and Adjusted 95% Confidence Interval for Various Measurements

Variables	MoCA	Age	Computer proficiency	Computer self-efficacy	Nature exposure	Initial attitude	Depression
MoCA		(–0.48, 0.29)	(−0.28, 0.49)	(−0.29, 0.47)	(−0.42, 0.31)	(−0.27, 0.29)	(−0.30, 0.45)
Age	−0.11		(−0.28, 0.35)	(−0.38, 0.31)	(−0.51, 0.28)	(−0.40, 0.31)	(−0.55, 0.25)
Computer proficiency	0.12	0.04		(0.22,0.80)	(−0.37,0.30)	(−0.25, 0.55)	(−0.28, 0.51)
Computer self-efficacy	0.10	−0.04	0.58**		(−0.52,0.27)	(−0.21, 0.58)	(−0.44, 0.30)
Nature exposure	−0.07	−0.13	−0.04	−0.15		(−0.53, 0.26)	(−0.17, 0.61)
Initial attitude	0.01	−0.05	0.18	0.22	−0.16		(−0.28, 0.50)
Depression	0.09	−0.18	0.14	−0.08	0.26	0.13	
Mean	25.88	67.97	25.91	56.86	26.60	70.18	32.25
*SD*	2.81	4.85	3.39	8.16	4.04	10.73	18.76

*Notes:* **Significant at the 0.001 level after Holm’s correction for multiple comparisons. MoCA = Montreal Cognitive Assessment; *SD* = standard deviation.

### Changes in Mood States (H1)

**Figure 4.**
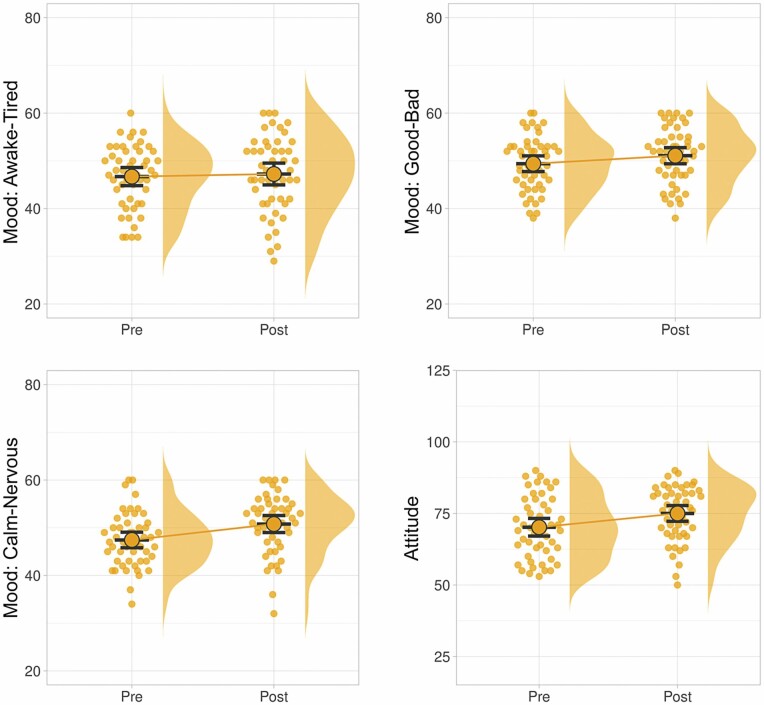
Changes in mood states and in attitudes toward VR technology before and after exposure to the virtual garden environment. Significant improvements were seen in all cases except for the awake/tired mood state. VR = virtual reality.

The first hypothesis in the study predicted that short-term exposure to the virtual environments would be associated with improvements in mood states. We calculated the change on each subdimension of the *Mood State Questionnaire* (good/bad mood, calm/nervous mood, and awake/tired mood) between the preimmersion and postimmersion participant responses. We used *t* tests to compare this change for the good/bad and calm/nervous dimensions and found that the both of these showed significant positive improvement after the VR experience. The data for the awake/tired dimension were found to violate normal-distribution assumptions, so a Wilcoxon signed-rank test was used to evaluate the change in this dimension. No significant change was found in awake/tired from preimmersion to postimmersion (Table 4 and Figure 4).

**Table 4.** Wilcoxon and *t* Tests for Changes in Mood and Attitude

Variables	*V* ^1^	*p*	*x̄* (*SD*)	*t*(49)^2^	*p*
Mood: awake/tired	613	.283			
Mood: calm/nervous			3.34 (6.17)	3.826	<.001
Mood: good/bad			1.68 (4.78)	2.486	.016
Attitude toward VR			4.82 (8.76)	3.892	<.001

*Note: SD* = standard deviation; VR = virtual reality.

### Changes in Attitude Toward VR Technology (H2)

The second study hypothesis predicted that short-term exposure to the virtual environments would be associated with improvements in attitudes toward VR technology. A one-sample *t* test confirmed this hypothesis based on a comparison of responses to the *Acceptance of Head-Mounted Virtual Reality* scale preimmersion and postimmersion (Table 4 and Figure 4).

### CI Versus Non-CI Participants (H3)

**Figure 5.**
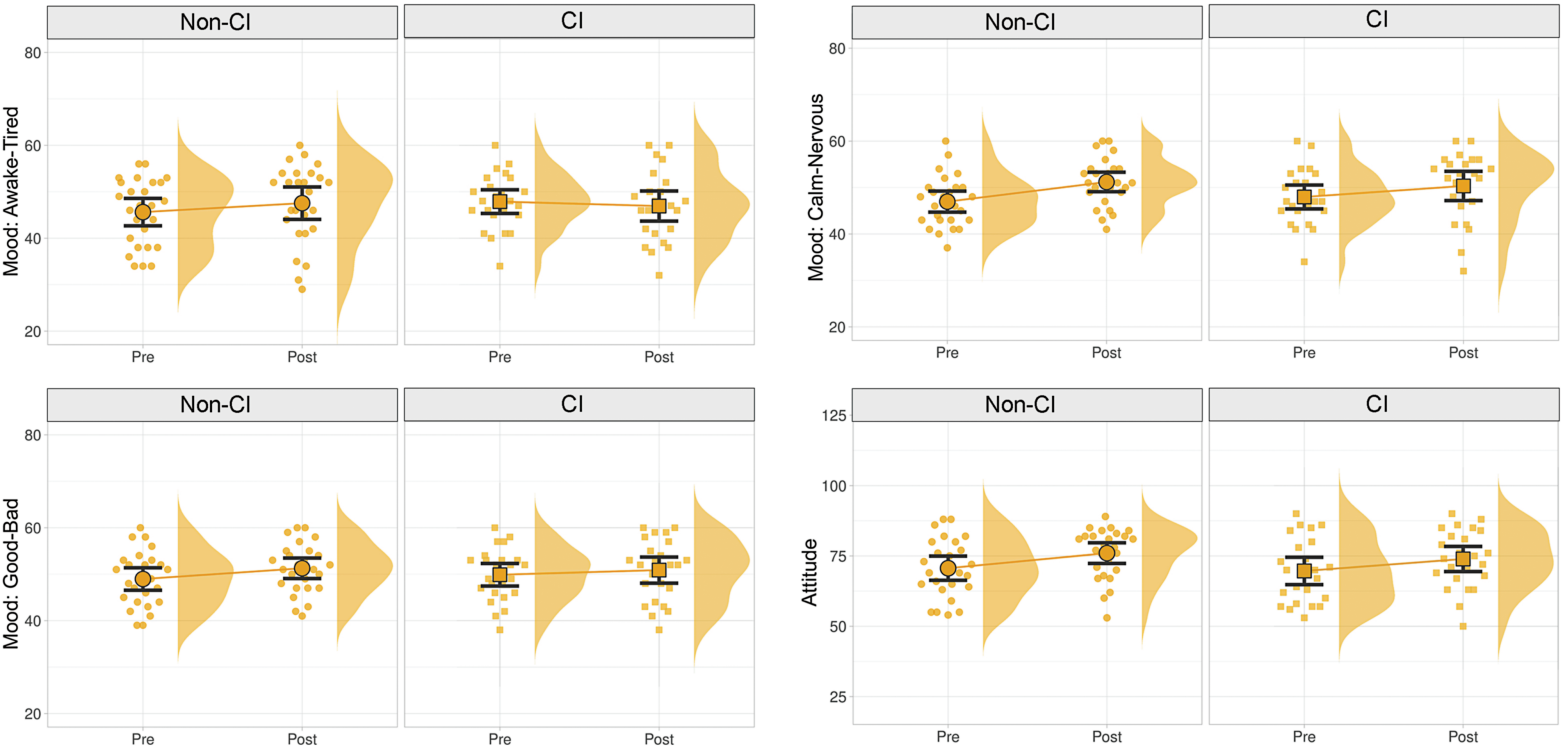
Changes in mood states and in attitudes toward VR technology before and after exposure to the virtual environment, compared between cognitive impairment (CI) and non-CI groups. There were no significant differences found in the changes seen between the CI participants versus the non-CI participants. VR = virtual reality.

The third hypothesis predicted that changes in mood states and attitudes toward VR would not differ significantly between participants with different cognitive abilities. We used participants’ scores on the *Montreal Cognitive Assessment* (MOCA) to divide them into a group with likely mild CI and a group without CI (this does not constitute a formal diagnosis, but the MOCA is a commonly used screening tool). A linear model was used to examine the effects of the CI grouping on the mood states and attitude variables while adjusting for age, education level, computer efficiency (both *Computer Proficiency* and *Computer Self-Efficacy* scores), and *Nature Exposure*. The results indicated no significant differences in the changes of mood and attitude between the CI and the non-CI groups (Table 5 and Figure 5).

**Table 5.** Difference Between CI and Non-CI Groups

Outcome	Difference	*SE*	95% CI	*t*	*p*	Cohen’s *d*
Mood: awake/tired (Delta)	−2.69	2.06	(−6.86, 1.47)	−1.306	.198	−0.380
Mood: calm/nervous (Delta)	−2.19	1.85	(−5.92, 1.54)	−1.184	.243	−0.344
Mood: good/bad (Delta)	−1.35	1.45	(−4.28, 1.57)	−0.932	.356	−0.271
Attitude toward VR (Delta)	−1.41	2.45	(−6.35, 3.53)	−0.575	.568	−0.167
Simulator sickness	−0.12	1.06	(−2.26, 2.02)	−0.114	.910	−0.033
NASA task load index	0.13	0.16	(−0.19, 0.45)	0.833	.410	0.242
Social desirability	−0.10	0.64	(−1.39, 1.19)	−0.156	.877	−0.045
Spatial presence: self-location (SPSL)	−1.18	1.62	(−4.44, 2.08)	−0.728	.471	−0.212
Spatial presence: possible actions (SPPA)	−1.04	1.36	(−3.77, 1.70)	−0.763	.450	−0.222
System usability scale	2.62	1.49	(−0.39, 5.62)	1.756	.086	0.511

*Notes:* df = 43. Difference = CI−non-CI. Adjusted for age, education level, computer proficiency, computer self-efficacy, and everyday nature exposure. CI = cognitive impairment; *SE* = standard error; VR = virtual reality.

### Participants With Physical Disabilities Versus Without Disabilities (H4)

The fourth hypothesis predicted that participants with physical disabilities would report more pronounced changes in mood states and attitudes toward VR after exposure to the virtual environment, compared to participants without disabilities. Physical disability status was self-reported on the survey instruments. Similar to the examination of CI status in Hypothesis 3, we fitted a linear model that examined the effects of disability on outcomes while adjusting for age, education level, *Computer Proficiency* scores, *Computer Self-Efficacy* scores, and *Nature Exposure* scores.

**Figure 6.**
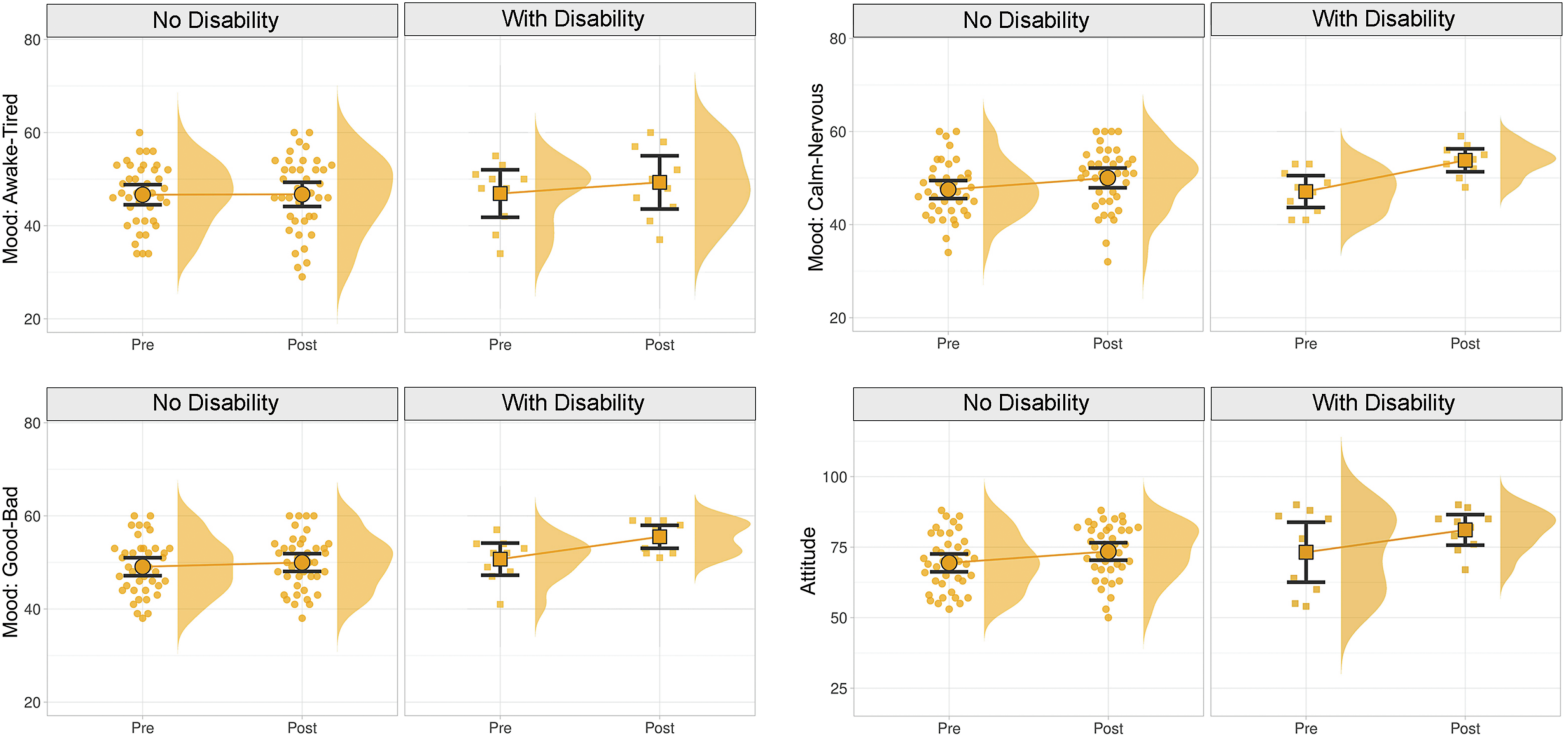
Changes in mood states and in attitudes toward VR technology before and after exposure to the virtual environment, compared between disability and nondisability groups. Significant differences were found between these two groups for the good/bad and calm/nervous mood dimensions (participants with disabilities responded more positively to the VR experience in these measures). No statistically significant differences were found between the disability versus nondisability groups for the awake/tired mood dimension or for changes of attitudes toward VR technology. VR = virtual reality.

The results indicated a significant difference in the “good/bad” mood dimension (*p* = .030), with a large effect size (*d* = 0.820), as well as a weakly significant difference in the calm/nervous mood dimension (*p* = .076) with a medium effect size (*d* = 0.665). In both cases, the participants with disabilities showed a larger shift toward positive outlooks, compared to the nondisability group. The awake/tired mood dimension and the attitudes toward VR technology did not show significant differences between participants with disabilities versus those without disabilities (Table 6 and Figure 6).

**Table 6.** Differences Between Disability and Nondisability Groups

Outcome	Difference	*SE*	95% CI	*t*	*p*	Cohen’s *d*
Mood: awake/tired (Delta)	2.16	2.62	(−3.13, 7.45)	0.824	.415	0.301
Mood: calm/nervous (Delta)	4.15	2.28	(−0.45, 8.74)	1.821	.076	0.665
Mood: good/bad (Delta)	3.91	1.74	(0.40, 7.43)	2.244	.030	0.820
Attitudetoward VR(Delta)	3.64	3.04	(−2.49, 9.77)	1.198	.237	0.438
Simulator sickness	−0.08	1.33	(−2.76, 2.61)	−0.059	.953	−0.022
NASA task load index	0.01	0.20	(−0.39, 0.41)	0.050	.960	0.018
Social desirability	−0.44	0.80	(−2.05, 1.17)	−0.555	.582	−0.203
Spatial presence: self-location (SPSL)	4.47	1.93	(0.58, 8.35)	2.318	.025	0.847
Spatial presence: possible actions (SPPA)	2.53	1.67	(−0.84, 5.91)	1.512	.138	0.552
System usability scale	1.78	1.92	(−2.09, 5.64)	0.927	.359	0.339

*Notes:* df = 43. Difference = disability−nondisability. Adjusted for age, education level, computer proficiency, computer self-efficacy, and everyday nature exposure. *SE* = standard error; VR = virtual reality.

### Qualitative Results

The interview transcripts were analyzed using Lincoln and Guba’s (1985) Naturalistic Inquiry method for interpreting qualitative data. This method involves separating the content from all participants into discrete segments, sorting the data into categories until saturation is achieved, and then summarizing the results. In regard to the overall VR experience, most of the participants indicated that the system was intuitive and easy to learn, and that the environment was relaxing and pleasant. In fact, the most common complaint about the VR experience was that the sessions ended too quickly. Several participants expressed feelings of nervousness about taking actions in the environment or about the visual perspectives they encountered, most notably regarding one of the nature videos that included a broad vista: “The one where you felt like you were really high … [that] made me really nervous and I was afraid to move about for fear of falling” (P09). Another negative reaction that was expressed by more than half of the participants was that the interactive digital components felt “cartoonish” or artificial after the experience of viewing the nature videos. Reactions to the 360-degree videos themselves were universally positive with no negative feedback. The participants frequently praised the realism of this component: “I felt like I was really right in it!” (P41). One participant noted that in the past they had frequently visited a site where we filmed one of the nature scenes: “Having stood in that exact spot on the hill it was … very, very close to real” (P29).

Approximately equal numbers of participants expressing a sense of full immersion versus a sense of being a detached observer or not really present in the environment. The most commonly cited obstacles to immersion were physical reminders of the real-world context, such as when the connecting cables of the headset brushed up against the participant’s body, or when they had to adjust their position due to moving too close to the edge of the VR floor-space. In addition, one participant indicated difficulties in focusing on the display that impacted immersion: “[The image] was just slightly out of focus … I still felt like I was there and I was just needing to focus my eyes a little differently” (P54). Another participant indicated that it felt awkward to wear glasses along with the head-mounted display. Several individuals also noted concerns about the hand-held controllers, indicating that the joystick was small and difficult to grasp and manipulate: “My hands are pretty arthritic—they don’t hurt and they can still move—but at some point what happens when people’s hands aren’t that mobile?” (P50). Another participant linked the issue of CIs with potential interface difficulties: “I think that people with mild cognitive impairments might have problems with the controls” (P41). These comments indicate technological design needs that are somewhat beyond the scope of the current study—it seems probable that VR platform manufacturers do not view older individuals as their targeted consumer market. If these technologies are to have therapeutic benefits for older adults, however, then at some point the issue of comfortable and accessible controls will need to be addressed.

A majority of the participants expressed appreciation for the sense of closeness to plants and animals and the ability to interact with them in the garden. While many indicated that the digital aspects did not feel entirely realistic, they nonetheless brought delight and engagement and were reminiscent of real-world memories: “I like that within the virtual world I was able to make choices, like picking flowers. I enjoyed the proximity of the flowers and the animals like the duckling” (P14). When asked about their willingness to engage in the experience again and their likelihood of recommending it to others, approximately three quarters of the participants responded positively. Many of the comments in this area focused on the value of the VR environment for individuals who lack the ability to experience real-world nature in-person, such as those with severe CI or limited mobility: “[The VR experience could] transport them out of the crappy situation they’ve been dealt, into a beautiful place” (P41). Several participants also posited that the VR experience would be useful during a pandemic, when opportunities to visit real-world natural areas were limited. However, a few participants remained skeptical of VR technology as a general concept, and were conflicted in their responses due to concerns that the VR environment would replace real-life experiences: “It’s hard to tell how therapeutic it would be to plant fake flowers in a fake garden. I don’t know, I can’t quite imagine being in a state where that would be enough, as opposed to just reminding [users] that they couldn’t go out into a real garden” (P45).

The positive comments about the utility of the VR environment often tended to focus on its role in prompting active engagement, and its potential uses for physical therapeutic purposes: “Absolutely, for people with impairments that are [getting use of] their hands back or whatever, I think it’s … a wonderful tool” (P43). “I can imagine it … keeping you intrigued and keeping you mentally stimulated as well as physically stimulated” (P53). “I think would be wonderful to keep an active mind and I think would be great idea” (P37). Notably, the majority of both positive and negative comments about the applicability of the platform tended to focus on its impacts for *other* users who were imagined to be in different circumstances than the speaker. This may have been due to hesitation on the part of participants to see themselves as a potential user of virtual environments, even if they had experienced benefits from their encounter with the technology.

## Discussion and Implications

Overall, the study findings indicated that the virtual environments were suitable for older adults with and without CIs. For most participants, the VR experience had a positive impact on mood and resulted in improved attitudes toward the technology. Very little cybersickness or frustration with the technology was reported. The general improvements in mood states after experiencing the VR are consistent with prior findings (Appel et al., 2020; Russell et al., 2013), and they support the theory that VR can serve as a restorative or stress-reducing environment. However, the current study findings do not provide evidence about the long-term impact of VR-use for mental health or cognitive function, which will need to be evaluated in further studies. The study also did not find improvements in the awake/tired dimension of the mood instrument—this may be due to the inherent fatigue involved in learning a new technology and from completing numerous questionnaires during the session. To some extent, there may be a trade-off between engagement levels and fatigue, and prior researchers have noted that VR technologies can be intrinsically tiring (Moyle et al., 2018; Siriaraya & Siang Ang, 2014; Thach et al., 2020). These issues need to be accounted for when considering session lengths and frequency of use. However, we did find significant improvements in reporting “good” mood and “calm” mood outcomes after the VR sessions, and many participants expressed heightened engagement and a desire to spend more time in the VR.

The improvements in attitudes toward VR are particularly notable, as older adults frequently exhibit hesitancy and negative feelings about novel technologies (Broady et al., 2010; Hauk et al., 2018). The current study indicated that even one brief session of VR can result in a significant shift in such attitudes toward more positive perceptions. The researchers speculate that further VR sessions would likely result in additional reductions in hesitancy, particularly if the regular use of the technology was well-integrated into broader everyday life and the experiences were shared with friends and family (Appel et al., 2020). The finding that changes in mood and attitude were not significantly different between those with and without a CI suggests that these interventions could be beneficial to individuals with declining cognitive capabilities. However, the current study did not evaluate the responses of individuals with more severe degrees of CI. There is strong evidence that novel experiences can incite fear and anxiety among older adults with advanced cognitive decline (Moyle et al., 2018), and thus particular caution should be used if introducing such individuals to VR. Adjustments to the experience, such as including human facilitators or guides, may be advisable for those with moderate to severe impairment. Reminiscence-based activities have been shown to be effective in improving mood and engagement (Chin, 2007; Hsieh & Wang, 2003; Manera et al., 2016), and indeed, in the interviews for the current study we found that several participants spontaneously remarked on how the environment brought to mind past experiences. Thus, the stability and consistency of the VR platform and a combination of familiar elements, family members, or locations with new and stimulating experiences may be important to consider in long-term therapeutic use.

The study findings indicated that virtual environments could be especially beneficial to individuals with mobility restrictions or other physical disabilities. The relatively greater improvements in mood and attitudes toward VR that were reported by participants with disabilities (compared to those without disabilities) may be grounded in the understanding that such individuals often confront severe accessibility issues and have fewer overall opportunities to engage with nature outside of the VR (Stigsdotter et al., 2018). Correspondingly, it might be conjectured that individuals will gain a greater benefit from VR if their broader circumstances limit their ability to spend time in nature, such as individuals who live in dense urban conditions or in harsh environmental climates. It is notable, however, that the current study did not find a correlation between the extent of everyday nature exposure and improvements in mood after using the VR. This finding may be due to the study participants having a consistently high level of real-world nature exposure, leading to a lack of statistical variability needed to produce a correlation. It may also be the case that the greater benefits reported by disabled participants are more closely related to specific accessibility features and comfort levels in real-world versus virtual environments, rather than to access to nature in general.

### Technological Considerations

While the majority of the participants indicated that the VR equipment was intuitive, comfortable, and easy to use (reflecting the overall improvements in attitudes toward VR after the experience), there were some specific technological concerns that arose during the interviews. One such concern was related to the visual perspectives presented in the nature videos, which incited feelings of precarity and/or fears of falling for some participants. This is an understandable reaction, as vertigo is a common human experience, and injury from falls is a particularly significant concern for older individuals (Fuller, 2000). This issue needs to be taken into account when filming immersive videos for restorative VR use, by ensuring that the perspectives of the videos are well-grounded in a stable and safe vantage point.

In regard to the digitally created interactive elements, many of the participants expressed a sense of disappointment in the level of graphical realism and sharpness. One participant noted: “I thought I’d be seeing the real world and be working in that, but I realize how can you do that when you have to create a world where you can pick something up so it can’t necessarily be, you know, pictures” (P43). To some extent, the level of realism in digital environments is related to the amount of time and resources that are available to create the renderings. Ongoing improvements in VR technologies, as well as additional time to create more detailed graphical elements for the virtual garden, will likely help to improve these participant reactions and increase the sense of immersion in the environment.

While the number of participants reporting discomfort or distraction with the VR equipment was not high, some important considerations were raised in the interviews about the small size of the joystick, distracting due to contact with the headset cables, and the awkward fit of the headset when wearing glasses. These are important material design considerations that should be taken into account when developing VR applications for older adults.

### Limitations and Future Research

The current study findings indicated significant enhancements in mood states and in attitudes toward VR technology after a 30-min multifaceted exploration experience. However, the mechanism under which the VR Garden created these positive outcomes needs further study. The current results do not definitively indicate that nature-related content was a deciding feature, as it may have been other aspects of VR engagement (encountering a novel technology, general interactive stimulus) that led to the reported benefits. The role of the natural components, and of specific environmental design features, needs to be investigated in future studies that compare nature-based VR against other types of VR content. Further attention to demographic variables in larger population samples may provide additional insights into how diverse users respond to different types of VR content.

We are currently working to update the virtual platform based on feedback from the study participants, with an emphasis on improving the digital garden with greater realism and a wider array of interactive features. New innovations in computer graphics and tactile technology, which are emerging almost daily, will likely lead to a higher degree of realism and immersion. One of our important short-term goals is to integrate social components into the environment, which we believe will help to enhance engagement and build bridges between the VR and real-world contexts. These features will allow selected friends, family members, caretakers, and peers to work collaboratively and to share and discuss their experiences in the VR. We are also preparing for future longitudinal studies that will evaluate the impact of regular use of the VR environment over time for mental health states such as depression and for the maintenance of cognitive function. This future work will likely separate the passive viewing of 360-degree nature videos and engagement with the digital garden into separate trials, in order to better isolate the impacts of these different forms of virtual experience.

## Supplementary Material

igac015_suppl_Supplementary_Material
