# Supplementary material for: Using a Nature-Based Virtual Reality Environment for Improving Mood States and Cognitive Engagement in Older Adults: A Mixed-Method Feasibility Study
Source: Innov Aging. 2022 Mar 17;6(3):igac015. doi: 10.1093/geroni/igac015 (PMC9113189; doi:10.1093/geroni/igac015)
Supplement: igac015_suppl_Supplementary_Material [file igac015_suppl_supplementary_material.docx]

**Supplementary Material**

**S1. Measurement Instruments**

In the demographic survey, the *Nature Exposure Scale II* was used to assess each participant’s exposure to nature in everyday life. This simple instrument consists of 6 items, each rated on a 5-point Likert scale, with 1=”low” and 5=”high” (Wood et al., 2019). The ratings on the 6 items were summed to obtain a total nature-exposure score with a possible range of 6–30. Also on the demographic survey, the participants’ likelihood of having depression was assessed using the *Center for Epidemiologic Studies Depression Scale* (Hann et al., 1999). This instrument consists of 10 items, each on a 4-point Likert scale, with positive items reverse coded (in other words, higher scores indicate a greater likelihood of having depression). Ratings on all of the items were summed, and participants with a total score greater than 16 were classified as likely to be suffering from some degree of depression.

Proficiency with computers was assessed in the initial demographic questionnaire using two scales: the *Computer Self-efficacy Scale* (Barbeite & Weiss, 2004) and the *Computer Proficiency Scale* (Boot et al., 2015). The first instrument focuses on confidence levels in relation to information technology, while the second instrument focuses on proficiency with different computer domains (printers, the Internet, entertainment software, etc.). Both instruments use 5-point Likert scales, with higher scores indicating greater proficiency.

Participants’ cognitive capabilities were evaluated at the time of the experiment using the *Montreal Cognitive Assessment* (Nasreddine et al., 2005). This instrument does not in itself confirm a diagnosis of cognitive impairment, but it is commonly used as a screening tool to assess cognitive status. The assessment involves several brief written and verbal tasks measuring executive functions, memory, language, and reasoning. In the current study the assessment was used to divide the participants into those with a likely cognitive impairment and those without a cognitive impairment, with a threshold score of lower than 26 on the instrument designated as likely-impaired. There has been some debate regarding the exact threshold score on this instrument that indicates likely cognitive impairment, but < 26 is the most commonly used metric (Carson et al., 2018; Milani et al., 2018; Wong et al., 2015). None of the participants in the current study scored low enough on this instrument to be regarded as having severe cognitive impairment.

The mood states of each participant were assessed immediately before and immediately after the VR session, using the *Multidimensional Mood State Questionnaire* (Steyer et al., 1997). This instrument includes 30 items, each on a 6-point Likert scale, with higher scores indicating more positive mood states. The results are divided into three dimensions, including “good/bad” mood (GB), “calm/nervous” mood (CN), and “awake/tired” mood (AT). The total scores of a participant for each of these three dimensions were calculated separately.

Participants’ attitudes toward VR technology were also assessed immediately before and immediately after the VR session. The instrument used for this purpose was a scale developed by Huygelier and colleagues (2019) for the specific purpose of evaluating *Acceptance of Head-mounted Virtual Reality in Older Adults*. It includes 18 items, each on a 5-point Likert scale, with higher scores indicating a more positive attitude toward the technology. The scores on all items were summed to obtain a total VR-attitude score.

Immersion levels were measured after the VR session using the *MEC Spatial Questionnaire* (Vorderer et al., 2004). This instrument is a multidimensional measure of spatial presence and its components. In this study, a 6-item scale on Spatial Presence: Possible Actions (SPPA), and a 6-item scale on Spatial Presence: Self Location (SPSL) subscales were employed.

Finally, two instruments were used after the VR session to measure potential negative impacts of the technology. Participants were asked to complete the *NASA Task Load Index* (Hart & Staveland, 1988) to evaluate perceptions of effort and frustration as related to engagement with the virtual environment. This instrument includes six subscales, including mental demand, physical demand, temporal demand, effort, performance, and frustration level, each on a 5-point Likert scale, with higher scores indicating greater perceived task loads. Total scores for each participant were obtained by summing the responses for all items. The *Simulator Sickness Questionnaire* (Kennedy, Lane, Berbaum, & Lilienthal, 2009) was used to assess experiences of “cybersickness.” This instrument includes 16 items on a 4-point Likert scale, ranging from “none” to “severe,” with higher scores indicating greater levels of discomfort. The responses on all items were summed to get a total cybersickness score for each participant.
